# Differential impact of asparaginase discontinuation on outcomes of children with T‐cell acute lymphoblastic leukemia and T‐cell lymphoblastic lymphoma

**DOI:** 10.1002/cam4.7246

**Published:** 2024-06-18

**Authors:** Hisashi Ishida, Toshihiko Imamura, Ryoji Kobayashi, Yoshiko Hashii, Takao Deguchi, Takako Miyamura, Megumi Oda, Masaki Yamamoto, Keiko Okada, Hideki Sano, Katsuyoshi Koh, Yuki Yuza, Kenichiro Watanabe, Noriyuki Nishimura, Tetsuya Takimoto, Akiko Moriya‐Saito, Masahiro Sekimizu, Souichi Suenobu, Shosuke Sunami, Keizo Horibe

**Affiliations:** ^1^ Department of Pediatrics Okayama University Hospital Okayama Japan; ^2^ Department of Pediatrics Kyoto Prefectural University of Medicine, Graduate School of Medical Science Kyoto Japan; ^3^ Department of Hematology/Oncology for Children and Adolescents Sapporo Hokuyu Hospital Sapporo Japan; ^4^ Department of Pediatrics Osaka International Cancer Institute Osaka Japan; ^5^ Division of Cancer Immunodiagnostics, Children's Cancer Center National Center for Child Health and Development Tokyo Japan; ^6^ Department of Pediatrics Osaka University Graduate School of Medicine Suita Japan; ^7^ Department of Pediatrics Sapporo Medical University School of Medicine Sapporo Japan; ^8^ Department of Pediatric Hematology/Oncology Osaka City General Hospital Osaka Japan; ^9^ Department of Pediatric Oncology Fukushima Medical University Hospital Fukushima Japan; ^10^ Department of Hematology/Oncology Saitama Children's Medical Center Saitama Japan; ^11^ Department of Hematology and Oncology Tokyo Metropolitan Children's Medical Center Tokyo Japan; ^12^ Department of Hematology and Oncology Shizuoka Children's Hospital Shizuoka Japan; ^13^ Department of Public Health Kobe University Graduate School of Health Science Kobe Japan; ^14^ Department of Childhood Cancer Data Management National Center for Child Health and Development Tokyo Japan; ^15^ Clinical Research Center National Hospital Organization Nagoya Medical Center Nagoya Japan; ^16^ Department of Pediatrics National Hospital Organization Nagoya Medical Center Nagoya Japan; ^17^ Department of Pediatrics Oita University Oita Japan; ^18^ Department of Pediatrics, Japanese Red Cross Narita Hospital Narita Japan

**Keywords:** acute pancreatitis, allergy, asparaginase, children, T‐cell acute lymphoblastic leukemia, T‐cell lymphoblastic lymphoma

## Abstract

**Background:**

Asparaginase is essential for treating T‐cell acute lymphoblastic leukemia (T‐ALL). Despite the ongoing debate on whether T‐ALL and T‐cell lymphoblastic lymphoma (T‐LBL) are the same disease entity or two distinct diseases, patients with T‐LBL often receive the same or similar treatment protocols as those with T‐ALL.

**Methods:**

The outcomes of patients with or without L‐asparaginase discontinuation were retrospectively analyzed among four national protocols: Japan Association of Childhood Leukemia Study (JACLS) ALL‐02 and ALL‐97 for T‐ALL and Japanese Pediatric Leukemia/Lymphoma Study Group ALB‐NHL03 and JACLS NHL‐98 for T‐LBL. The hazard ratio (HR) was calculated with the Cox regression model by considering L‐asparaginase discontinuation as a time‐dependent variable.

**Results:**

In total, 199 patients with T‐ALL, and 133 patients with T‐LBL were included. L‐asparaginase discontinuation compromised event‐free survival (EFS) of T‐ALL patients (ALL‐02: HR 3.32, 95% confidence interval [CI] 1.40–7.90; ALL‐97: HR 3.39, 95%CI 1.19–9.67). Conversely, EFS compromise was not detected among T‐LBL patients (ALB‐NHL03: HR 1.39, 95%CI 0.41–4.68; NHL‐98: HR 0.92, 95%CI 0.11–7.60).

**Conclusion:**

The effects of L‐asparaginase discontinuation differed between T‐ALL and T‐LBL. We assume that the differential impact results from (1) the inherent differential response to L‐asparaginase between them and/or (2) a less stringent assessment of early treatment response in T‐LBL than in T‐ALL. Given the poor salvage rate of refractory or relapsed T‐ALL and T‐LBL, optimization of the frontline therapy is critical, and the current study provides a new suggestion for further treatment modifications. However, larger studies in contemporary intensified treatment protocols are required.

## Graphical abstract

The outcomes of patients with or without L‐asparaginase discontinuation were retrospectively analyzed among four national protocols, two with T‐cell acute lymphoblastic leukemia (T‐ALL) and two with T‐cell lymphoblastic lymphoma (T‐LBL). The effects of L‐asparaginase discontinuation differed between T‐ALL and T‐LBL, and given a poor salvage rate of refractory or relapsed T‐ALL and T‐LBL, optimization of the frontline therapy is critical, and the current study provided a new suggestion for further treatment modifications.

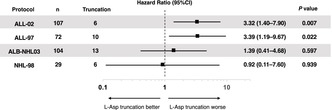

## 1 INTRODUCTION

Asparaginase plays an important role in the chemotherapeutic regimens to treat acute lymphoblastic leukemia (ALL). However, asparaginase is frequently discontinued due to its unique adverse effects, such as clinical allergy, subclinical hypersensitivity, acute pancreatitis, and thrombosis.^1^, ^2^ Asparaginase has been thought to be even more effective and important for T‐cell ALL than for B‐cell ALL,^3^, ^4^ and we have previously identified that L‐asparaginase discontinuation was associated with compromised outcomes among children with T‐ALL.^5^ However, our previous study analyzed only one protocol and included 79 patients with T‐ALL who achieved remission after induction therapy; thus, external validation would be warranted.

The discussion about whether T‐ALL and T‐cell lymphoblastic lymphoma (T‐LBL) are a spectrum of the same disease or two distinct disease entities is ongoing. Remarkable similarities have been suggested regarding clinical or cytogenetic characteristics,^6^ and the World Health Organization and the International Lymphoma Study Group classify these two diseases into one entity, that is, T‐lymphoblastic leukemia/lymphoma.^7^, ^8^ As such, children with T‐LBL have received the same or similar treatment protocols as those with T‐ALL.^9^, ^10^, ^11^, ^12^, ^13^, ^14^ On the contrary, several recent clinical and biological data raised the possibility that T‐ALL and T‐LBL are different disease entities.^15^ They differ in terms of immunophenotypes,^16^ gene expression profiles,^17^, ^18^ and DNA methylation characteristics.^19^ Furthermore, patients with cancer predisposition syndromes predispose to each of T‐ALL or T‐LBL specifically. Ataxia telangiectasia predisposes to T‐ALL and mature B‐cell non‐Hodgkin lymphomas but rarely to T‐LBL.^20^, ^21^ Conversely, Nijmegen breakage syndrome predisposes to lymphomas including T‐LBL but less frequently to T‐ALL.^22^ In this context, the effect of discontinuation of asparaginase may differ between these two diseases, and the impact of asparaginase discontinuation in children with T‐LBL should be investigated as well, and should be compared to those with T‐ALL.

Here, we analyzed the characteristics and outcomes of patients with or without L‐asparaginase discontinuation among four national protocols for T‐ALL and T‐LBL: Japan Association of Childhood Leukemia Study (JACLS) ALL‐02 and ALL‐97 for T‐ALL and Japanese Pediatric Leukemia/Lymphoma Study Group (JPLSG) ALB‐NHL03 and JACLS NHL‐98 for T‐LBL.

## 2 PATIENTS AND METHODS

### 2.1 Patients

The current study included clinical data of patients with T‐ALL or advanced stage (III or IV) T‐LBL extracted from four treatment protocols: JACLS ALL‐02, JCACLS ALL‐97, JACLS NHL‐98, and JPLSG ALB‐NHL03 (UMIN000002212, http://www.umin.ac.jp/ctr/index‐j.htm).^5^, ^23^, ^24^, ^25^ The JACLS ALL‐02 study, conducted between 2002 and 2008, included 107 patients with T‐ALL. The JACLS ALL‐97 study, conducted between 1997 and 2001, included 72 patients with T‐ALL. The JPLSG ALB‐NHL03 study, conducted between 2004 and 2010, included 104 patients with T‐LBL (68 patients with stage III and 36 patients with stage IV disease). The JACLS NHL‐98 study, conducted between 1998 and 2002, included 29 patients with T‐LBL (19 patients with stage III and 10 patients with stage IV disease). Accordingly, we included those with T‐ALL and T‐LBL who were treated in similar years. Each patient's guardian provided informed consent per the Declaration of Helsinki. The Ethical Committees of all participating institutions approved the trials. Further information on the treatment details is available elsewhere.^5^, ^23^, ^24^, ^25^ Previously, we investigated the effects of L‐asparaginase discontinuation on patients treated with JACLS ALL‐02 protocols^5^; in that study, we analyzed patients with T‐ALL who showed <25% blast at day15 bone marrow, achieved remission after induction, and thus received treatment based on the T‐02 protocol. On the contrary, in the current study, we analyzed all patients with T‐ALL.

### 2.2 Treatment protocols

The details and results of these four protocols have been previously published,^5^, ^23^, ^24^, ^25^ and the details are also summarized in Tables S1–S4 and Figure S1. L‐asparaginase schedules of each protocol are described as follows. L‐asparaginase was the only preparation of asparaginase approved for use in Japan during the study periods of these four protocols, and Erwinia asparaginase (Erwinase) could not replace the native L‐asparaginase in case of discontinuation. In all four protocols, L‐asparaginase was discontinued in case of systemic allergy reaction, acute pancreatitis, or asparaginase‐related complications for which the treating physician decided to discontinue asparaginase. These reasons and the timing of asparaginase discontinuation were centrally reported. Subclinical hypersensitivity was not measured in these clinical studies. Additional drugs were administered only in the ALL‐02 protocol after L‐asparaginase discontinuation. No treatment modification was applied besides L‐asparaginase discontinuation in the other protocols. The total doses of L‐asparaginase of these protocols are summarized in Table 1.

**TABLE 1:** The cumulative doses of L‐asparaginase according to the treatment protocols.

Protocol	Cumulative L‐asparaginase (U/m^2^)	Premaintenance (U/m^2^)	Maintenance (U/m^2^)
ALL‐02 (T‐02)	246,000	66,000	180,000
ALL‐97 (T‐97)	600,000	260,000	340,000
ALB‐NHL03	234,000	90,000	144,000
NHL‐98	600,000	260,000	340,000

JACLS ALL‐02: Among patients with T‐ALL, those who showed blast <25% at Day 15 bone marrow and achieved remission after induction received treatment based on T‐02 protocols (*n* = 84), while those who failed to achieve above criteria were allocated to F protocol (*n* = 23). The induction phase comprised six doses of L‐asparaginase (6000 U/m^2^ per dose) administered for 12 days. In T‐02 protocol, patients received L‐asparaginase therapy (6000 U/m^2^ per dose) for 5 consecutive days during the sanctuary therapy. They also received two courses of six doses of intermittent L‐asparaginase (10,000 U/m^2^) and two courses of three doses of L‐asparaginase (10,000 U/m^2^) for 14 days during the maintenance therapy. If L‐asparaginase was discontinued, patients received one course of intensified therapy, including high‐dose cytarabine, etoposide, and PSL, immediately before the initiation of maintenance therapy. In F protocol, after induction phase, patients received two courses of consolidation therapy, each containing five doses of L‐asparaginase (6000 U/m^2^). JACLS ALL‐97 and JACLS NHL‐98: Both protocols applied the same schedules of L‐asparaginase therapy as described previously.^23^ Among patients with T‐ALL, those who showed blast <5% at Day 15 bone marrow and achieved remission after induction received treatment based on T‐97 protocols (*n* = 61), while those who failed to achieve above criteria were allocated to F protocol (*n* = 11). In summary, in ALL‐97 and NHL‐98 protocols, the induction phase comprised six doses of L‐asparaginase (10,000 U/m^2^ per dose) administered for 12 days, followed by consolidation therapies A and B, which both contained five doses of L‐asparaginase (10,000 U/m^2^ per dose) administered for 5 consecutive days. Patients received consolidation therapies A and B twice. During the maintenance therapy, patients received block B thrice, block C twice, and block D thrice. Block C comprised five doses of L‐asparaginase (10,000 U/m^2^ per dose) administered for 5 consecutive days. Blocks B and D contained three L‐asparaginase doses (10,000 U/m^2^ per dose) administered for 14 days. JPLSG ALB‐NHL03: The induction phase comprised nine doses of L‐asparaginase (6000 U/m^2^ per dose) administered for 19 days. During the reinduction therapy, six doses of L‐asparaginase (6000 U/m^2^ per dose) were administered for 12 days. A total of fourteen doses of L‐asparaginase (6000 U/m^2^ per dose) were administered during the early maintenance therapy, and 10 doses (6000 U/m^2^ per dose) were administered during the late maintenance therapy.

### 2.3 Statistical analysis

Event‐free survival (EFS) and overall survival (OS) were defined as the time from diagnosis to the date of an event, where events were defined as death from any cause for OS and as first relapse, death, induction failure, or secondary neoplasm for EFS. Induction failure was defined as patients who did not achieve <5% blasts in the bone marrow for those with T‐ALL and those who did not achieve complete remission (CR), unconfirmed remission, or partial response for T‐LBL. Induction failure was defined as an event at the predefined evaluation dates after the induction therapy: day 33 for JACLS ALL‐02 and JPLSG ALB‐NHL03 and day 35 for JACLS ALL‐97 and NHL‐98. Competing events were defined as death without relapse for relapse and relapse for nonrelapse mortality. Hazard ratio (HR) for EFS was calculated using extended Cox regression model, and HR for incidence of relapse was calculated using Fine and Gray competing risk regression model by considering asparaginase discontinuation as a time‐dependent variable.^26^ Statistical analyses were conducted using EZR (Saitama Medical Center, Jichi Medical University, Saitama, Japan),^27^ a graphical user interface for R (The R Foundation for Statistical Computing, Vienna, Austria). *p* < 0.05 was considered statistically significant for all analyses.

## 3 RESULTS

### 3.1 Patient characteristics

Patient characteristics across four treatment protocols are described in Table 2. Sex distribution was similar; however, the age at diagnosis was lower in patients with T‐ALL (median 8 years for T‐ALL vs. 10 years for T‐LBL, *p* < 0.01). The rate of induction failure was similar (7.3% for T‐ALL vs. 6.8% for T‐LBL, *p* = 1.00). Median follow‐up periods for survivors in each protocol were 77.6 (range, 29.2–129.3) months for JACLS ALL‐02, 122.5 (range, 60.4–167.4) months for JACLS ALL‐97, 63.2 (range, 33.4–95.4) months for JPLSG ALB‐NHL03, and 109.7 (range, 46.7–157.2) months for JACLS NHL‐98.

**TABLE 2:** Patient characteristics.

	T‐ALL			T‐LBL			*p* Value
	ALL‐02	ALL‐97	Total T‐ALL	ALB‐NHL03	NHL‐98	Total T‐LBL	
	*n* = 107	*n* = 72	*n* = 179	*n* = 104	*n* = 29	*n* = 133	
Mean age (range)	8.0 (1–15)	9.5 (2–15)	8.0 (1–15)	10.0 (1–15)	11.0 (3–14)	10.0 (1–15)	<0.001
Sex							
Male	88 (82.2%)	49 (68.1%)	137 (76.5%)	78 (75.0%)	24 (82.8%)	102 (76.7%)	1.000
Female	19 (17.8%)	23 (31.9%)	42 (23.5%)	26 (25.0%)	5 (17.2%)	31 (23.3%)	
CNS infiltration							
Positive	10 (9.3%)	1 (1.4%)	11 (6.1%)	4 (3.8%)	1 (3.4%)	5 (3.8%)	0.440
Negative	97 (90.6%)	71 (98.6%)	168 (93.9%)	100 (96.2%)	28 (96.6%)	128 (96.2%)	
Induction Failure							
Yes	9 (8.4%)	4 (5.6%)	13 (7.3%)	4 (3.8%)	5 (17.2%)	9 (6.8%)	1.000
No	98 (91.6%)	68 (94.4%)	166 (92.7%)	100 (96.2%)	24 (82.8%)	124 (93.2%)	

Abbreviations: ALL, acute lymphoblastic leukemia; LBL, lymphoblastic lymphoma.

The reasons for L‐asparaginase discontinuation are shown in Table 3, with acute pancreatitis being the most frequent as observed in 16/179 (6.7%) patients with T‐ALL and 16/133 (12.0%) patients with T‐LBL. Discontinuation due to allergy or thrombosis was rare, as only seen in <3% of patients in each protocol. The majority of patients discontinued L‐asparaginase before the start of the maintenance therapy phase, except for those in the NHL‐98 protocol (Table S5).

**TABLE 3:** The reasons for the discontinuation of L‐asparaginase.

	Patient number	Discontinuation number	Allergy	Pancreatitis	Thrombosis	Others
T‐ALL total	179	16 (8.9%)	1 (0.6%)	12 (6.7%)	2 (1.1%)	1 (0.6%)
ALL‐02	107	6 (5.6%)	1 (0.9%)	2 (1.9%)	2 (1.9%)	1 (0.9%)
ALL‐97	72	10 (13.9%)	0	10 (13.9%)	0	0
T‐LBL total	133	19 (14.3%)	3 (2.6%)	14 (10.5%)	3 (2.6%)	0
ALB‐NHL03	104	13 (12.5%)^a^	3 (2.9%)	8 (7.7%)	3 (2.9%)	0
NHL‐98	29	6 (20.7%)	0	6 (20.7%)	0	0

^a^ One patient discontinued L‐Asparaginase due to both acute pancreatitis and thrombotic event.

Abbreviations: ALL, acute lymphoblastic leukemia; LBL, lymphoblastic lymphoma.

### 3.2 Survival analysis

The 5‐year EFS of patients with T‐ALL was 54.4% (95% confidence interval (CI), 44.4%–63.4%) for JACLS ALL‐02 and 72.2% (95% CI, 60.3%–81.1%) for JACLS ALL‐97, and the 5‐year EFS of patients with T‐LBL was 76.9% (95% CI, 67.6%–83.9%) for JPLSG ALB‐NHL03 and 58.6% (95% CI, 38.8%–74.0%) for JACLS NHL‐98. The 5‐year OS of patients with T‐ALL was 72.7% (95% CI, 62.7–80.5%) for JACLS ALL‐02 and 81.9% (95% CI, 70.9%–89.1%) for JACLS ALL‐97, and the 5‐year OS of patients with T‐LBL was 80.4% (95% CI, 71.3%–86.9%) for JPLSG ALB‐NHL03 and 72.2% (95% CI, 85.1%–85.0%) for JACLS NHL‐98.

**FIGURE 1:**
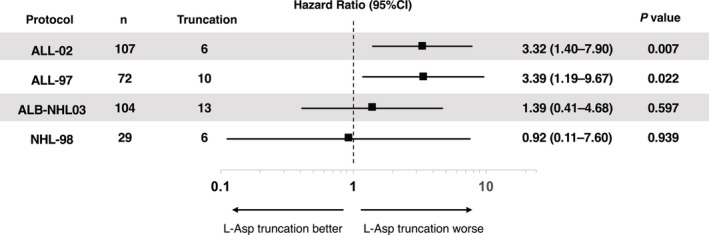
Hazard ratio of L‐asparaginase discontinuation on event‐free survival according to the treatment protocols. CI, confidence interval; L‐Asp, L‐Asparaginase.

**FIGURE 2:**
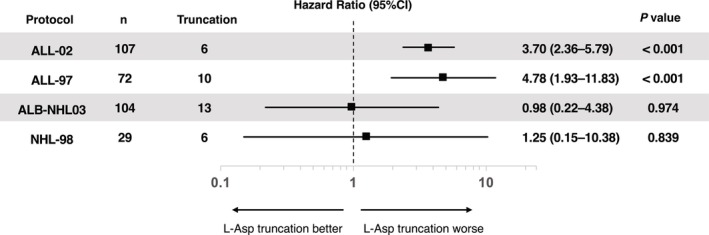
Hazard ratio of L‐asparaginase discontinuation on cumulative incidence of relapse according to the treatment protocols. CI, confidence interval; L‐Asp, L‐Asparaginase.

The HR was calculated by considering L‐asparaginase discontinuation as a time‐dependent variable. As shown in Figure 1, EFS was compromised when L‐asparaginase was discontinued in the JACLS ALL‐02 (HR 3.32, 95% CI 1.40–7.90) and JACLS ALL‐97 (HR 3.39, 95% CI 1.19–9.67) protocols. Conversely, EFS compromise was not detected in the JPLSG ALB‐NHL03 (HR 1.39, 95% CI 0.41–4.68) and JACLS NHL‐98 (HR 0.92, 95% CI 0.11–7.60) protocols. These results were maintained in the analysis for cumulative incidence of relapse (CIR). L‐asparaginase discontinuation was associated with worse CIR in T‐ALL patients, but not as clear in T‐LBL patients (Figure 2). The overall trend was less evident when the HR for OS was calculated (Figure S2).

## 4 DISCUSSION

Although the patient outcomes were significantly compromised when L‐asparaginase was discontinued in the two independent protocols for T‐ALL, the outcome was not altered when L‐asparaginase was discontinued in the two independent protocols for T‐LBL. This result is consistent with the recent evidence demonstrating differential responses to chemotherapy.^12^, ^13^, ^14^, ^28^ Given the poor salvage rate of refractory or relapsed T‐ALL and T‐LBL,^11^ optimization of the frontline therapy is critical, and the current study provided a new suggestion for further treatment modifications.

Our previous study using the cohort of JACLS ALL‐02 study showed that those with T‐ALL suffered compromised outcomes when L‐asparaginase was discontinued.^5^ In the current study, we expanded the inclusion criteria in the JACLS ALL‐02 protocol cohort and also referred to the independent ALL‐97 cohort. As shown in Figure 1, the compromised outcomes of those with T‐ALL were observed in both JACLS ALL‐02 and ALL‐97 cohorts. The background characteristics of the included patients in the two studies generally aligned with previous studies. The frequencies of central nervous system involvement at diagnosis were also similar to those of a previous study.^6^ Moreover, the total doses of L‐asparaginase in JACLS ALL‐97 were larger than those of ALL‐02 (Table 1), which we believed further supported the detrimental effect of L‐asparaginase discontinuation. As such, we assumed that we successfully confirmed the result of our previous study, where those with T‐ALL suffered compromised outcomes when L‐asparaginase was discontinued.^5^

The most striking result in the current study was the different effects of L‐asparaginase discontinuation between patients with T‐ALL and T‐LBL. In contrast to T‐ALL, the effect of L‐asparaginase discontinuation was much less clear in patients with T‐LBL in the current study. This was in line with the recent large clinical studies of the Children's Oncology Group (COG), highlighting differential responses to chemotherapy between T‐ALL and T‐LBL. In the COG AALL0434 and AALL1231 studies, those with T‐ALL and T‐LBL received similar treatment protocols, in which the researcher investigated the effects of adding newer agents, such as nelarabine or bortezomib.^12^, ^13^, ^14^ Interestingly, nelarabine improved the survival of patients with T‐ALL but not of those with T‐LBL in the AALL0434 trial. Meanwhile, bortezomib improved the survival of patients with T‐LBL but not of those with T‐ALL in the AALL1231 trial. These results suggested that T‐ALL and T‐LBL have different responses to specific treatment drugs, and we suspected that the response to L‐asparaginase might also be different between T‐ALL and T‐LBL. Furthermore, the study suggests the potential for varying effects of replacement therapies following asparaginase discontinuation between T‐ALL and T‐LBL, as children with T‐ALL would benefit more from such therapies.

Another possible explanation for this differential effect of L‐asparaginase discontinuation might be the difference in assessment measures of treatment responses between these two diseases. Among patients with T‐ALL, the response is measured by prednisone response, bone marrow morphology, or, more recently, polymerase chain reaction‐based, or flow‐cytometry‐based minimal residual disease detection. However, the response is usually measured by the tumor size changes evaluated with computed tomography in T‐LBL, and the stage at diagnosis is the only parameter for risk group stratification, classifying patients into limited (stage I and II) versus advanced (stage III and IV) stages. Accordingly, the evaluation of treatment response in T‐LBL might not be as stringent as in T‐ALL, and patients with T‐LBL who had suboptimal treatment response might continue to be included in the first‐line protocol without any intensification and would experience relapse subsequently, with or without L‐asparaginase discontinuation. Meanwhile, a recent study investigating the utility of fluoro‐deoxy‐glucose positron emission tomography (PET‐CT) showed a significant positive correlation between minimal residual disease and patient outcomes among those with ALL and LBL.^29^ Identifying patients with early suboptimal treatment responses using a more refined method such as PET‐CT^30^ might be critically needed for those with T‐LBL, but this should be evaluated in a prospective clinical trial.

As shown in Table 3, the incidence of acute pancreatitis differed based on treatment protocols. Asparaginase‐associated pancreatitis occurs in 2%–18% of patients treated for ALL,^31^, ^32^, ^33^, ^34^, ^35^, ^36^ and a previous study by Liu et al. identified older age and higher cumulative doses of asparaginase as risk factors of asparaginase‐associated acute pancreatitis.^33^ As shown in Tables 1 and 3, our study also demonstrated that the frequency of acute pancreatitis in protocols with higher doses of L‐asparaginase (JACLS ALL‐97; 13.9% and JACLS NHL‐98; 27.6%) seemed higher than that of protocols with lower doses (JACLS ALL‐02; 1.9% and JPLSG ALB‐NHL03; 7.7%), regardless of disease types. The frequency of L‐asparaginase discontinuation due to allergy was low among those receiving ALL‐02, while the frequency among those receiving ALL‐97 was similar to those of other countries.^37^ We attributed this difference in treatment schedules, and this was already discussed in our previous report.^5^

This study has several limitations. First, four different protocols were included. These differences make it difficult to directly compare the effects of L‐asparaginase discontinuation between T‐ALL and T‐LBL, as the differential effect of discontinuing asparaginase could depend more on protocol differences rather than on biological differences between T‐ALL and T‐LBL. In fact, in the NHL‐98 protocol, L‐asparaginase was discontinued more often during maintenance therapy than during the premaintenance phase (Table S5). This could pose a challenge in identifying the detrimental effects of L‐asparaginase discontinuation. Moreover, due to data limitations, exact doses before L‐asparaginase discontinuation could not be analyzed, hindering comparison among the four studies. However, it is worth mentioning that the detrimental effect of L‐asparaginase discontinuation was not observed among those who received the ALB‐NHL03 protocol, in which most patients discontinued L‐asparaginase before the start of the maintenance therapy. We are considering applying the same or similar treatment for T‐ALL and T‐LBL in Japan and expect that this strategy will allow us to directly compare the effects of asparaginase discontinuation. Second, as the survival outcomes of our protocols for T‐ALL were suboptimal compared to the contemporary protocols from other cooperating groups,^12^, ^13^, ^14^, ^38^, ^39^ the effect of asparaginase discontinuation might differ in the context of contemporary treatment protocols. Third, L‐asparaginase was the only preparation approved for use in Japan during the study period of these protocols, and the results might be different when using PEG‐asparaginase, which is much more widely used nowadays. Finally, we think the statistical results in this manuscript should be evaluated with caution. Initially, we attempted to analyze the effect of discontinuation using a landmark‐based method, as we did in the previous study. However, this did not seem feasible because many events occurred within the treatment period for those with T‐LBL in this study, making it difficult to set a suitable landmark for them. As our conclusion was based solely on one method,^26^ we suggest that the results should be further evaluated in external cohorts.

In conclusion, while the patient outcomes were significantly compromised when L‐asparaginase was discontinued in the two independent protocols for T‐ALL, the outcome did not differ when L‐asparaginase was discontinued in the two independent protocols for T‐LBL. We assumed that this differential impact of L‐asparaginase discontinuation resulted from (1) the inherent differential response to L‐asparaginase between T‐ALL and T‐LBL and/or (2) less strict assessment of early treatment response among T‐LBL than in T‐ALL. Given a poor salvage rate of refractory or relapsed T‐ALL and T‐LBL, optimization of the frontline therapy is critical, and the current study provided a new suggestion for further treatment modifications. Further studies with larger patient numbers in contemporary intensified treatment protocols are required to confirm this differential impact between T‐ALL and T‐LBL.

## AUTHOR CONTRIBUTIONS

**Hisashi Ishida:** Conceptualization (equal); formal analysis (equal); methodology (equal); writing – original draft (lead). **Toshihiko Imamura:** Conceptualization (equal); formal analysis (equal); methodology (equal); writing – review and editing (lead). **Ryoji Kobayashi:** Data curation (equal); writing – review and editing (supporting). **Yoshiko Hashii:** Data curation (equal); writing – review and editing (supporting). **Takao Deguchi:** Data curation (equal); writing – review and editing (supporting). **Takako Miyamura:** Data curation (supporting); writing – review and editing (supporting). **Megumi Oda:** Investigation (equal); writing – review and editing (supporting). **Masaki Yamamoto:** Investigation (equal); writing – review and editing (supporting). **Keiko Okada:** Investigation (equal); writing – review and editing (supporting). **Hideki Sano:** Investigation (equal); writing – review and editing (supporting). **Katsuyoshi Koh:** Investigation (equal); writing – review and editing (supporting). **Yuki Yuza:** Investigation (equal); writing – review and editing (supporting). **Kenichiro Watanabe:** Investigation (equal); writing – review and editing (supporting). **Noriyuki Nishimura:** Investigation (equal); writing – review and editing (supporting). **Tetsuya Takimoto:** Data curation (equal); writing – review and editing (supporting). **Akiko Moriya‐Saito:** Data curation (equal); writing – review and editing (supporting). **Masahiro Sekimizu:** Data curation (equal); formal analysis (supporting); writing – review and editing (supporting). **Souichi Suenobu:** Data curation (supporting); formal analysis (supporting); writing – review and editing (supporting). **Shosuke Sunami:** Data curation (equal); formal analysis (equal); project administration (equal); writing – review and editing (supporting). **Keizo Horibe:** Data curation (equal); funding acquisition (lead); project administration (equal); writing – review and editing (supporting).

## FUNDING INFORMATION

The JACLS ALL‐02 study and JPLSG ALB‐NHL03 studies were supported by Grants for Clinical Cancer Research from the Ministry of Health, Labor and Welfare of Japan.

## CONFLICT OF INTEREST STATEMENT

The authors have no conflicts of interest.

## ETHICS STATEMENT

The Ethical Committees of all participating institutions, including the institutional ethics committee of Kyoto Prefectural University of Medicine, approved the trials included in this study.

## PATIENT CONSENT STATEMENT

Each patient's guardian provided informed consent as per the Declaration of Helsinki.

## Supporting information


Appendix S1.

## Data Availability

The data that support the findings of this study are available from the corresponding author upon reasonable request.
