# Supplementary material for: Differential impact of asparaginase discontinuation on outcomes of children with T‐cell acute lymphoblastic leukemia and T‐cell lymphoblastic lymphoma
Source: Cancer Med. 2024 Jun 18;13(12):e7246. doi: 10.1002/cam4.7246 (PMC11184648; doi:10.1002/cam4.7246)
Supplement: Supplementary file 1 — Appendix S1. [file CAM4-13-e7246-s001.docx]

**Supplementary Data**

**Supplemental Table S1**. JACLS ALL T-02 treatment protocol

| Treatment element/drug | Single or daily dose | Days of application per element^a^ |
| --- | --- | --- |
| Pre-phase |  |  |
| Prednisolone | 60 mg/m^2^/d | 1–7 |
| Methotrexate | 12 mg/dose IT^b^ | 1 |
|  |  |  |
| Induction |  |  |
| Vincristine | 1.5 mg/m^2^ iv | 8, 15, 22, 29 |
| Pirarubicin | 20 mg/m^2^ div over 1 h | 8, 9 |
| L-asparaginase | 6,000 IU/m^2^ div over 4 h or im | 15, 17, 19, 22, 24, 26 |
| Cyclophosphamide | 1200 mg/m^2^ div over 1 h | 10 |
| Dexamethasone | 10 mg/m^2^ div over 1 h | 8–14 |
| Prednisolone | 40 mg/m^2^ po or div over 1 h | 15–28 and taper^d^ |
| TIT^c^ |  | 8, 22^e^ |
|  |  |  |
| Consolidation |  |  |
| Cyclophosphamide | 500 mg/m^2^ div over 1 h | 36, 38, 40 |
| Pirarubicin | 25 mg/m^2^ div over 1 h | 36, 37 |
| Cytarabine | 100 mg/m^2^ cont iv | 36–42 |
| Dexamethasone | 10 mg/m^2^ div over 1 h | 36–42 |
| TIT^c^ |  | 36, 43 |
|  |  |  |
| Sanctuary |  |  |
| Methotrexate | 3,000 mg/m^2^ div over 24 h | 64, 71 |
| Leucovorin | 15 mg/m^2^ iv x 6 q6 h | Start at 42 h from the beginning of methotrexate |
| Cytarabine | 100 mg/m^2^ cont iv | 72–76 |
| L-asparaginase | 6,000 IU/m^2^ div over 4 h or im | 73–77 |
| Prednisolone | 40 mg/m^2^ po | 71–77 |
| TIT^c^ |  | 65, 72 |
|  |  |  |
| Re-consolidation |  |  |
| Cyclophosphamide | 500 mg/m^2^ div over 1 h | 99, 101, 103 |
| Pirarubicin | 25 mg/m^2^ div over 1 h | 99, 100 |
| Cytarabine | 100 mg/m^2^ cont iv | 99–105 |
| Dexamethasone | 10 mg/m^2^ div over 1 h | 99–105 |
| TIT^c^ |  | 99, 106 |
|  |  |  |
| Early maintenance^f^ |  |  |
| Vincristine | 1.5 mg/m^2^ iv | 1, 22, 43 |
| Pirarubicin | 25 mg/m^2^ div over 1 h | 1, 22 |
| L-asparaginase | 10,000 IU/m^2^ im | 1, 8, 22, 29, 43, 50 |
| Prednisolone | 60 mg/m^2^ po | 1–5, 22–26, 43–47 |
| 6-mercaptopurine | 50 mg/m^2^ po | 1–14, 22–35, 43–56 |
| TIT^c^ |  | 1 |
| Repeat this therapy 2 times |  |  |
|  |  |  |
| Maintenance IA/IB |  |  |
| 6-mercaptopurine | 50 mg/m^2^ po | 1–28, 71–98 |
| Methotrexate | 150 mg/m^2^ po | 1, 15, 29, 71, 85, 99 |
| Vincristine | 1.5 mg/m^2^ iv | 43, 50, 57, 113, 120, 127 |
| Cyclophosphamide | 600 mg/m^2^ div over 1 h | 50, 120 |
| L-asparaginase | 10,000 IU/m^2^ im | 43, 50, 57, 113, 120, 127 |
| Prednisolone | 40 mg/m^2^ po | 43–57, 113–127 |
| TIT^c^ |  | 29 |
| Maintenance II |  |  |
| 6-mercaptopurine | 50 mg/m^2^ po | 1–28 |
| Methotrexate | 25 mg/m^2^ po | 1, 8, 15, 22 |
| Repeat this therapy 12 times  Intensification therapy  for those discontinued L-asparaginase^g^ |  |  |
| Pirarubicin | 40 mg/m^2^ div over 1 h | 1 |
| Etoposide | 150 mg/m^2^ div over 3 h | 2–4 |
| Cytarabine | 2,000 mg/m^2^ div over 3 h × 6 q12 h | 2–4 |
| Prednisolone | 40 mg/m^2^ po | 1–5 |
|  |  |  |

^a^Time schedule adjustments were allowed if clinical condition and bone marrow recovery were inadequate.

^b^Doses of IT drugs were adjusted for children < 3 years of age.

^c^TIT: intrathecal therapy with methotrexate, cytarabine, and hydrocortisone.

^d^Steroid doses were tapered over 7 additional days.

^e^Additional IT therapy was administered to patients with CNS3 status on days 11 and 15.

^f^For patients with CNS3 status or initial white blood cell count ≥100,000 /μL, 12 Gy cranial irradiation (1.5 Gy x 8 fractions) from day 1–12 and TIT on days 1 and 8 were administered. All intrathecal chemotherapy after irradiation was omitted.

^g^For those who discontinued L-asparaginase before the start of maintenance therapy, intensification chemotherapies were administered before maintenance therapy. For those who discontinued L-asparaginase during early maintenance therapy, maintenance therapy was stopped, and intensification chemotherapies were administered, and then maintenance therapy was resumed. For those who discontinued L-asparaginase after the start of maintenance IA, no additional therapy was administered.

Abbreviations: JACLS = Japan Association of Childhood Leukemia Study; div = intravenous infusion by drip; IT = intrathecal therapy; iv = intravenous infusion; im = intramuscular infusion; po = per oral; cont iv = continuous intravenous infusion; CNS = central nervous system; Fr = fraction.

**Supplemental Table S2**. JACLS ALL F protocol

| Treatment element/drug | Single or daily dose | Days of application per element^a^ |
| --- | --- | --- |
| Pre-phase |  |  |
| Prednisolone | 60 mg/m^2^/d | 1–7 |
| Methotrexate | 12 mg/dose IT^b^ | 1 |
|  |  |  |
| Induction |  |  |
| Vincristine | 1.5 mg/m^2^ iv | 8, 15, 22, 29 |
| Pirarubicin | 20 mg/m^2^ div over 1 h | 8, 9 |
| L-asparaginase | 6,000 IU/m^2^ div over 4 h or im | 15, 17, 19, 22, 24, 26 |
| Cyclophosphamide | 1200 mg/m^2^ div over 1 h | 10 |
| Dexamethasone | 10 mg/m^2^ div over 1 h | 8–14 |
| Prednisolone | 40 mg/m^2^ po or div over 1 h | 15–28 and taper^d^ |
| TIT^c^ |  | 8, (11^e^), (15^e^), 22 |
|  |  |  |
| Re-induction |  |  |
| Mitoxantrone | 8 mg/m^2^ div over 1 h | 1–3 |
| Cytarabine  Prednisolone  Etoposide  TIT^c^ | 500 mg/m^2^ div over 24 h  40 mg/m^2^ po  200 mg/m^2^ div over 4 h | 1–3, 8–10  1–3, 8–10  8–10  1 |
| Consolidation A |  |  |
| Mitoxantrone | 4 mg/m^2^ div over 1 h | 1–3 |
| Etoposide | 100 mg/m^2^ div over 2 h | 1–5 |
| Cytarabine | 100 mg/m^2^ div over 24 h | 1–5 |
| Dexamethasone | 100 mg/m^2^ div over 1 h | 1, 3, 5 |
| TIT^c^ |  | 1, (8^e^) |
|  |  |  |
| Consolidation B |  |  |
| Vincristine | 1.5 mg/m^2^ iv (max 2 mg) | 1 |
| Methotrexate  Cytarabine  L-asparaginase  Pirarubicin | 3,000 mg/m^2^ div over 24 h  100 mg/m^2^ div over 24 h  10,000 IU/m^2^ im  25 mg/m^2^ div over 1 h | 1  2–6  3–7  2 |
| Prednisolone | 40 mg/m^2^ po | 1–7 |
| TIT^c^ |  | 1, (8^e^) |
| Repeat consolidation A + B two times. |  |  |
| Maintenance Block 1 | Week 27, 37, 58, 57, 67, 77, 87, 97 |  |
| Methotrexate | 150 mg/m^2^ iv | 1, 15, 29 |
| 6-mercaptopurine | 50 mg/m^2^ po | 1–28 |
| TIT^c^ |  | 29 |
|  |  |  |
| Maintenance Block 2 | Week 33, 53, 93 |  |
| Vindesine | 3 mg/ m^2^ (max 4 mg) | 1, 8, 15 |
| Pirarubicin | 25 mg/m^2^ div over 1 hr | 8 |
| Etoposide | 100 mg/m^2^ div over 2 hr | 8 |
| Prednisolone | 40 mg/m^2^ po | 1–14 |
| Maintenance Block 3 | Week 43, 83, 103 |  |
| Vindesine | 3 mg/ m^2^ (max 4 mg) | 1, 8, 15 |
| Pirarubicin  Cyclophosphamide | 25 mg/m^2^ div over 1 hr  600 mg/m^2^ div over 1 hr | 8  8 |
| Prednisolone | 40 mg/m^2^ po | 1–14 |
|  |  |  |

Patients obtaining complete remission by the end of the first consolidation therapy were scheduled to receive hematopoietic stem cell transplantation after the second consolidation therapy but before maintenance therapy (i.e., after the fourth

consolidation therapy). Transplant procedures depended on the institute. After the fourth consolidation therapy maintenance chemotherapy was recommended for patients without a suitable transplantation donor or for whom transplantation was declined by the parents or guardians.

^a^Time schedule adjustments were allowed if clinical condition and bone marrow recovery were inadequate.

^b^Doses of IT drugs were adjusted for children < 3 years of age.

^c^TIT: intrathecal therapy with methotrexate, cytarabine, and hydrocortisone.

^d^Steroid doses were tapered over 7 additional days.

^e^Additional IT therapy was administered to patients with CNS3 status.

^f^For patients with CNS3 status or initial white blood cell count ≥100,000 /μL, 12 Gy cranial irradiation (1.5 Gy x 8 fractions) from day 1–10 and TIT on days 1 and 8 were administered. All intrathecal chemotherapy after irradiation was omitted.

Abbreviations: JACLS = Japan Association of Childhood Leukemia Study; div = intravenous infusion by drip; IT = intrathecal therapy; iv = intravenous infusion; im = intramuscular infusion; po = per oral.

**Supplemental Table S3**. JACLS ALL T-97 and NHL T-98 treatment protocol for T patients ^a^

| Treatment element/drug | Single or daily dose | Days of application per element^b^ |
| --- | --- | --- |
| Induction |  |  |
| Vincristine | 1.5 mg/m^2^ iv | 1, 8, 15, 22, 29, 36 |
| Cyclophosphamide  Adriamycin  L-asparaginase  Prednisolone  Prednisolone  Dexamethasone  TIT^d^  Consolidation therapy A  Vincristine  Methotrexate  Cytarabine  L-asparaginase  Prednisolone  TIT^d^  Consolidation therapy B  Vincristine  Cyclophosphamide  Adriamycin  L-asparaginase  Prednisolone  TIT^d^  Consolidation therapy A  Consolidation therapy B  Maintenance Block A  Methotrexate  6-mercaptopurine  Maintenance Block B  Vincristine  Cyclophosphamide  L-asparaginase  Prednisolone  Maintenance Block C  Vincristine  Methotrexate  Cytarabine  L-asparaginase  Pirarubicin  Prednisolone  TIT^d^  Maintenance Block D  Vincristine  Cyclophosphamide  L-asparaginase  Prednisolone | 1,200 mg/m^2^ 1h div  25 mg/m^2^ 1h div  10,000 IU/m^2^ 4h div or im  40 mg/m^2^/day 1 h div  40 mg/m^2^/day po  10 mg/m^2^ 1hdiv  1.5 mg/m^2^ iv  3,000 mg/m^2^ div over 24 h  100 mg/m^2^/day cont iv  10,000 IU/m^2^ 4h div or im  40 mg/m^2^/day po  1.5 mg/m^2^ iv  1,200 mg/m^2^ 1 h div  25 mg/m^2^ 1h div  10,000 IU/m^2^ 4h div or im  40 mg/m^2^/day po  150 mg/m^2^ iv  50 mg/m^2^ po  1.5 mg/m^2^ iv  600 mg/m^2^ 1 h div  10,000 IU/m^2^ 4h div or im  40 mg/m^2^/day po  1.5 mg/m^2^ iv  1,000 mg/m^2^ div over 24 h  100 mg/m^2^/day cont iv  10,000 IU/m^2^ im  25 mg/m^2^ 1 h div  40 mg/m^2^/day po  1.5 mg/m^2^ iv  600 mg/m^2^ 1 h div  10,000 IU/m^2^ 4h div or im  40 mg/m^2^/day po | 1, 29  2, 4, 30, 32  9, 11, 13, 16, 18, 20  -2 to 0  8–28, and tapering  1–7  1, 15, 29, 36  1  1  2–6  3–7  1–7  2  1, 8  1  2, 4  3–7  1–9  1  1, 15  1–21  1  8  1, 8, 15  1–15  1  1  2–6  3–7  2  1–7  2  1, 8, 15  8  1, 8, 15  1–15 |

Maintenance was done as follows:

A-B-A-C-A-B-A-C-A-B-A-D-A-B-A-D-A-B-A-D

^a^In ALL T-97 protocol, cranial irradiation was adapted for patients with WBC over 50 x10^9^/L at initial diagnosis. Except this point, ALL T-97 and NHL T-98 protocol were quite same.

^b^Time schedule adjustments were allowed if clinical condition and bone marrow recovery were inadequate.

^c^Time schedule adjustments were allowed if clinical condition and bone marrow recovery were inadequate.

^d^TIT: intrathecal therapy with methotrexate, cytarabine, and hydrocortisone.

Abbreviations: JACLS = Japan Association of Childhood Leukemia Study; div = intravenous infusion by drip; IT = intrathecal therapy; iv = intravenous infusion; im = intramuscular infusion; po = per oral; cont iv = continuous intravenous infusion

**Supplemental Table S4**. JPLSG ALB-NHL03 protocol

| Treatment element/drug | Single or daily dose | Days of application per element^a^ |
| --- | --- | --- |
| Induction/consolidation protocol I  Phase A |  |  |
| Prednisolone  Prednisolone  Vincristine | 30 mg/m^2^/day po or iv  60 mg/m^2^/day po or iv  1.5 mg/m^2^ iv | 1–3  4–28  8, 15, 22, 29 |
| Daunorubicin  L-asparaginase  Cyclophosphamide  TIT^b^  Phase B  Cyclophosphamide  Cyclophosphamide  Cytarabine  6-mercaptopurine  TIT^b^  Protocol M  6-mercaptopurine  Methotrexate  TIT^b^  Reinduction, protocol II  Dexamethasone  Vincristine  Cyclophosphamide  Daunorubicin  L-asparaginase  Cytarabine  6-mercaptopurine  TIT^b^  Early maintenance  Methotrexate  Prednisolone  Vincristine  Cyclophosphamide  Pirarubicin  L-asparaginase  6-mercaptopurine  TIT^b^  Repeat this therapy 2 times  Late maintenance  Prednisolone  Vincristine  Methotrexate  6-mercaptopurine  L-asparaginase  TIT^c^  Repeat this therapy 5 times | 40 mg/m^2^ 1h div  6,000 IU/m^2^ iv or im  1,000 mg/m^2^ 1h div  1,000 mg/m^2^ 1h div  500 mg/m^2^ 1h div  75 mg/m^2^/day iv  60 mg/m^2^ po  25 mg/m^2^ po  5,000 mg/m^2^ div over 24 h  10 mg/m^2^ po  1.5 mg/m^2^ iv  1,000 mg/m^2^ 1h div  40 mg/m^2^ 1h div  6,000 IU/m^2^ iv or im  75 mg/m^2^/day iv  60 mg/m^2^ po  500 mg/m^2^ 6h div  40 mg/m^2^/day po  1.5 mg/m^2^ iv  500 mg/m^2^ 1 h div  25 mg/m^2^ 1h div  6,000 IU/m^2^ iv or im  60 mg/m^2^ po  40 mg/m^2^/day po  1.5 mg/m^2^ iv  150 mg/m^2^ div over 6 h  60 mg/m^2^ po  6,000 IU/m^2^ iv or im | 8, 15, 22  9, 11, 13, 16, 18, 20, 23, 25, 27  8  1, (8)^c^, 15, (23)^c^, 9  36  50  36–39, 43–46, 50–53, 57–60  36–63  36, 50  1–35  1, 15, 29  1, 15, 29  1–14  1, 8, 15, 22  1, 29  1, 8, 15  9, 11, 13, 16, 18, 20  29–32, 36–39  29–42  1, 15, 29, 43^d^  1  15–19, 36–40, 57–61  15, 36, 57  15, 36  57  2, 15, 22, 36, 43, 57, 64  15–77  1  43–47  43, 50  1, 15, 29  1–56  43, 50  1 (first cycle only) |

^a^Time schedule adjustments were allowed if clinical condition and bone marrow recovery were inadequate.

^b^TIT: intrathecal therapy with methotrexate, cytarabine, and hydrocortisone.

^c^For CNS-positive patients.

^d^For CNS-negative patients.

**Supplemental Table S5**. Treatment phase of L-asparaginase discontinuation

| Protocol | Total truncation | pre-maintenance | maitenance |
| --- | --- | --- | --- |
| ALL-02 | 6 | 5 (83.3%) | 1 (16.7%) |
| ALL-97 | 10 | 9 (90.0%) | 1 (10.0%) |
| ALB-NHL03 | 13 | 8 (61.5%) | 5 (38.5%) |
| NHL-98 | 8 | 2 (25.0%) | 6 (75.0%) |

**Supplementary Figure S1.**

**Supplementary Figure S1.** Overview of treatment protocols and schedules of L-asparaginase.

**Abbreviations**: JACLS, Japan Association of Childhood Leukemia Study; JPLSG, Japanese Pediatric Leukemia/Lymphoma Study Group; Consol, consolidation; Re-ind, re-induction.

**Supplementary Figure S2.**


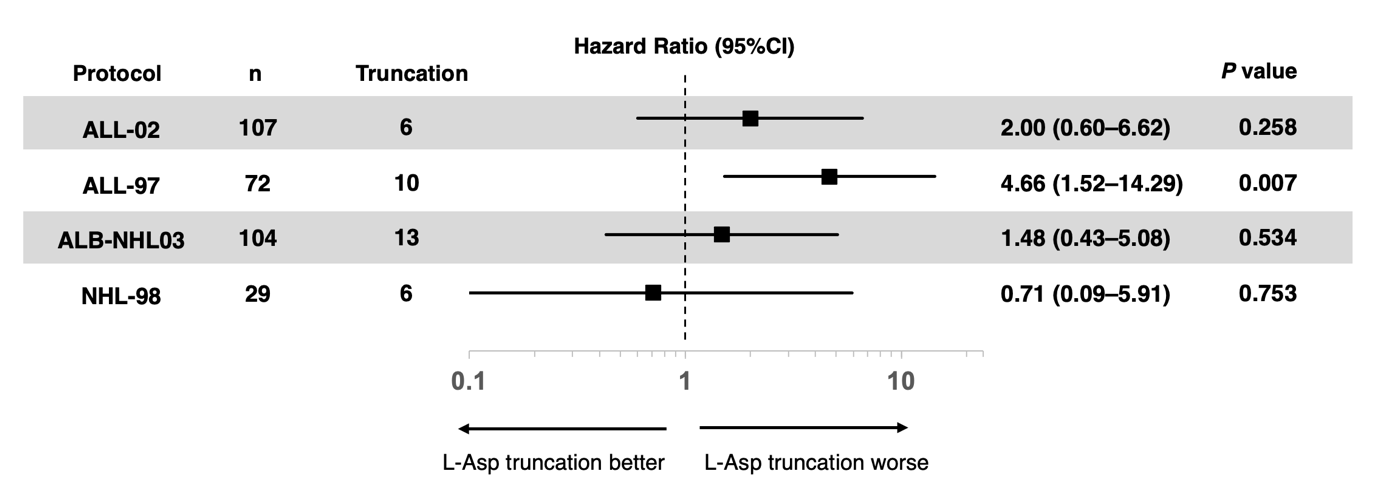


**Supplementary Figure S2.** Hazard ratio of the discontinuation of L-asparaginase on overall survival according to the treatment protocols.

**Abbreviations**: L-Asp, L-Asparaginase; CI, confidence interval.
